# *YBX1* gene silencing inhibits migratory and invasive potential via *CORO1C* in breast cancer *in vitro*

**DOI:** 10.1186/s12885-017-3187-7

**Published:** 2017-03-16

**Authors:** Jia Pei Lim, Sukanya Shyamasundar, Jayantha Gunaratne, Olivia Jane Scully, Ken Matsumoto, Boon Huat Bay

**Affiliations:** 1 Department of Anatomy, Yong Loo Lin School of Medicine, National University of Singapore, 4 Medical Drive, Blk MD10, Singapore, 117594 Singapore; 2 Quantitative Proteomics Group, Institute of Molecular and Cell Biology, Agency for Science, Technology and Research, 61 Biopolis Drive, Proteos, Singapore, 138673 Singapore; 3 Laboratory of Cellular Biochemistry, RIKEN, 2-1 Hirosawa, Wako, Saitama, 351-0198 Japan

**Keywords:** YB-1 protein, *CORO1C* gene, Migration, Invasion, Metastasis, Breast cancer

## Abstract

**Background:**

Y-box binding protein-1 is an evolutionary conserved transcription and translation regulating protein that is overexpressed in various human malignancies, including breast cancer. Despite reports of YB-1 and its association with distant spread of breast cancer, the intrinsic mechanism underlying this observation remains elusive. This study investigates the role of YB-1 in mediating metastasis in highly invasive breast cancer cell lines.

**Methods:**

Silencing the *YBX1* gene (which encodes the YB-1 protein) by small interfering RNA (siRNA) was performed in MDA-MB-231 and Hs578T breast cancer cell lines, followed by phenotypic assays including cell migration and invasion assays. Gene expression profiling using Affymetrix GeneChip® Human Transcriptome 2.0 array was subsequently carried out in YB-1 silenced MDA-MB-231 cells. Overexpression and silencing of *YBX1* were performed to assess the expression of *CORO1C*, one of the differentially regulated genes from the transcriptomic analysis. A Gaussia luciferase reporter assay was used to determine if *CORO1C* is a putative YB-1 downstream target. siRNA-mediated silencing of *CORO1C* and down-regulation of *YBX1* in *CORO1C* overexpressing MDA-MB-231 cells were performed to evaluate cell migration and invasion.

**Results:**

Downregulation of the YB-1 protein inhibited cell migration and invasion in MDA-MB-231 breast cancer cells. Global gene expression profiling in the *YBX1* silenced MDA-MB-231 cells identified differential expression of several genes, including *CORO1C* (which encodes for an actin binding protein, coronin-1C) as a potential downstream target of YB-1. While knockdown of *YBX1* gene decreased *CORO1C* gene expression, the opposite effects were seen in YB-1 overexpressing cells. Subsequent verification using the reporter assay revealed that *CORO1C* is an indirect downstream target of YB-1. Silencing of *CORO1C* by siRNA in MDA-MB-231 cells was also observed to reduce cell migration and invasion. Silencing of *YBX1* caused a similar reduction in *CORO1C* expression*,* concomitant with a significant decrease in migration in Hs578T cells. In coronin-1C overexpressing MDA-MB-231 cells, increased migration and invasion were abrogated by YB-1 knockdown.

**Conclusion:**

It would appear that YB-1 could regulate cell invasion and migration *via* downregulation of its indirect target coronin-1C. The association between YB-1 and coronin-1C offers a novel approach by which metastasis of breast cancer cells could be targeted and abrogated.

**Electronic supplementary material:**

The online version of this article (doi:10.1186/s12885-017-3187-7) contains supplementary material, which is available to authorized users.

## Background

Breast cancer is the leading cancer that affects women around the world, where the majority of deaths due to this dreaded disease could be attributed to metastasis. The World Health Organisation (WHO) has ranked breast cancer as the most common cause of cancer-related deaths in women in 2012, accounting for approximately 14.3% of cancer-related mortality in less developed countries [1]. Metastasis involves the invasion of cancer cells from the primary tumour site to the surrounding extracellular matrix and stroma, from wherein the cancer cells intravasate, travel through the vasculature and extravasate to form a secondary tumour at a distant site [2]. It is estimated that approximately 10–15% of breast cancer patients, show evidence of distant metastasis within 3 years from the initial detection of the primary tumour [3]. However, in some breast cancer patients, metastasis occurs after 10 years from the initial presentation of the primary tumour [4]. Furthermore, the heterogeneous nature of breast cancer makes it difficult for identification of patients who are at risk of developing metastasis.

Recent research has shed light on a potential biomarker for early metastasis, namely Y-box binding protein-1 (YB-1) encoded by the *YBX1* gene. YB-1 is an evolutionary conserved protein with a cold-shock domain, and is crucial to many fundamental cellular processes, including transcription and translation regulation [5]. Elevated YB-1 has been observed in many human malignancies, such as prostate cancer [6], gastric cancer [7, 8] and nasopharyngeal cancer [9]. YB-1 overexpression has been found be an independent prognostic marker in breast cancer [10]. Overexpression of YB-1 in the mammary gland of a novel transgenic mouse model showed that YB-1 induced genetic instability, leading to breast cancer [11]. In addition, YB-1 is involved in the upregulation of the transcription of multidrug resistance 1 (*MDR1*) gene which encodes for P-glycoprotein, a protein which mediates chemoresistance [12, 13]. YB-1 also contributes to enhanced membrane type I-matrix metalloproteinase (MT1-MMP) activity in MCF7 breast cancer cells, thus inducing tumor invasion and metastasis [14]. Moreover, YB-1 has been observed to potentiate epithelial to mesenchymal transition (EMT), possibly through the elevated translation of Snail1, which is an important mediator of the EMT process, and eventually leading to enhanced metastasis [15].

This study attempts to elucidate the role of YB-1 in mediating the metastatic cascade, through manipulation of YB-1 expression in aggressive MDA-MB-231 and Hs578T breast cancer cells. Gene expression profiling revealed that knockdown of YB-1, inhibited migratory and invasive potential with altered expression of several genes, in invasive MDA-MB-231 cells. Functional annotation clustering using the DAVID analysis, demonstrated that these differentially expressed genes are important for the cytoskeletal pathway, including *CORO1C* which encodes coronin-1C, an actin-binding protein. siRNA mediated silencing of *CORO1C* in MDA-MB-231 cells was observed to decrease cell migration and invasion (similar to YB-1 silenced cells). Similar findings were also observed in Hs578T breast cancer cells. Furthermore, transient overexpression of coronin-1C resulted in increased cell migration and invasion, which was abrogated by YB-1 knockdown in MDA-MB-231 cells. We show for the first time that YB-1 could regulate cell invasion and migration, possibly *via* regulation of its downstream target coronin-1C.

## Methods

### Cell culture

The human MDA-MB-231 breast cancer cell line (ATCC® HTB-26™) was cultured in RPMI 1640 medium, which contained 10% fetal bovine serum (FBS). Hs578T breast cancer cells (ATCC® HTB-126™) were propagated in DMEM medium with 10% FBS and supplemented with 50 μg/ml insulin (Sigma-Aldrich, St. Louis, MO, USA).

### Short interfering RNA (siRNA) transfection

2.5 × 10^5^ MDA-MB-231 cells and 1 × 10^5^ Hs578T cells were seeded in each well of a 6-well plate, a day prior to siRNA transfection. The ON-TARGETplus SMARTpool siRNA (GE Dharmacon, Pitssburgh, PA, USA), consisting of 4 individual siRNA targeting *YBX1* or *CORO1C* were used. A non-targeting siRNA was used as the negative control (*siNeg*). Triplicate wells were seeded for each of the siRNA. The transfection was carried out following the manufacturer’s protocol. Briefly, a final concentration of 20 nM for each of the siRNA was added together with the transfection reagent DharmaFECT (GE Dharmacon). The cells, together with the transfection mixture were incubated for 24 h, after which they were replenished with fresh medium. Cells were grown for 48 h and 72 h after transfection for RNA extraction and protein extraction respectively.

### Quantitative real-time polymerase chain reaction (qPCR)

After extraction of total RNA with the RNeasy Mini extraction kit (Qiagen, Hilden, Germany), 1 μg of total RNA was converted to first strand cDNA using the SuperScript III First-Strand synthesis system (Invitrogen, Carlsbad, CA, USA). Gene expression of *YBX1* and *CORO1C* were subsequently quantified in an Applied Biosystems 7900HT Fast Real-Time PCR system using the Fast SYBR Green Master Mix (Applied Biosystems, Foster City, CA, USA). Each sample was run in triplicates and *GAPDH* was used as the housekeeping gene for normalisation. Alterations in gene expression were expressed as fold change using the 2^-∆∆CT^ method [16]. The primers used are listed in Additional file 1: Table S1.

### Western blot

Whole cell lysates were extracted using a mixture of radio-immunoprecipitation (RIPA) lysis buffer (Pierce, Waltham, MA, USA), Halt Protease and Phosphatase Inhibitor Cocktail (Pierce) and 0.5 ethylenediaminetetraacetic acid (EDTA) (Pierce) on ice for 15 min. After cell lysis, the lysates were centrifuged for 15 min at 21,000 g, 4 °C. Protein concentration was determined using the microtiter Bio-Rad Protein Assay (Bio-Rad, Hercules, CA, USA). The same amount of protein lysates were denatured at 95 °C in 5X loading dye for 5 min and loaded into each well of the 10% sodium dodecyl sulfate-polyacrylamide gel. After separation by electrophoresis, proteins were transferred to a polyvinyl difluoride (PVDF) membrane using a semi-dry system (Bio-Rad). Transfer of proteins was carried out at 20 V for 1 h. Blocking of the PVDF membrane was subsequently done using 5% skim milk, followed by incubation with primary antibodies that include anti-coronin-1C (1:1000 dilution) (Abnova, Taipei City, Taiwan), anti-HIPK3 (1:5000) (Abcam, Cambridge, UK), anti-SLAIN2 (1:1000 dilution) (Abcam), anti-YB-1 (1:1000 dilution) [9] and anti-β-actin (1:6000 dilution) (Sigma-Aldrich) at 4 °C overnight. Subsequently, incubation with the HRP-conjugated secondary antibody (Sigma-Aldrich) was carried out for 1 h at room temperature. The SuperSignal West Pico Chemiluminescent ECL substrate (Pierce) was used to detect the protein bands which were then quantified using the GS-800 densitometer (Bio-Rad).

### Cell migration and cell invasion assays

Transwell migration assay was performed by using polycarbonate membrane transwell inserts (Corning, NY, USA), with a membrane diameter of 8.0 μm. A BD BioCoat™ Matrigel™ Invasion Chamber (BD Biosciences, San Jose, CA, USA) with an 8 μm pore size Polyethylene Terephthalate membrane, was used for the cell invasion assay. 48 h post-transfection with siRNA, 20,000 cells/well or 30,000 cells/well were seeded in triplicates in the upper chamber for the transwell migration or invasion respectively for MDA-MB-231 cells. For Hs578T cells, 10,000 cells/well and 20,000 cells/well were seeded and incubated for 18 h or 20 h, for the migration and invasion assays respectively. After which, the migratory or invasive cells were fixed with absolute methanol for 15 min, washed twice and stained with 0.5% crystal violet in water for 20 min. Removal of excess crystal violet stain was carried out by dipping the upper chamber inserts in distilled water and wiped away with cotton swabs from the upper membrane of the inserts. A Nikon SMZ 1500 stereo microscope at 10X magnification was subsequently used to view the stained cells. Five different fields were imaged and the average number of cells per insert was counted. Experiments were performed in triplicates.

### Gene microarray

To determine potential targets of YB-1 in MDA-MB-231 cells, global gene expression profiling was performed using Affymetrix GeneChip® Human Transcriptome 2.0 Array (Affymetrix, Santa Clara, CA, USA). Briefly, 100 ng of RNA was reverse transcribed to generate double stranded cDNA that was amplified to produce cRNA. The cRNA generated was purified and subjected to 2nd –cycle single stranded sense cDNA that was fragmented, labelled and hybridized to Affymetrix GeneChip® Human Transcriptome 2.0 array. The arrays were washed, stained and scanned using the Affymetrix 3000 7G scanner. The differentially expressed genes between *siNeg* and *siYB-1* transfected cells were determined using a fold change cut-off at 2 and *p* < 0.05 with the Transcriptome Analysis Console 3.0 software and annotation file HTA-2_0.na36.hg19.transcript.csv available from Affymetrix. Functional annotation clustering of the differentially expressed genes was carried out using the Database for Annotation, Visualization and Integrated Discovery (DAVID).

### Transfection of YB-1 overexpression plasmid

A cell density of 2.5 × 10^5^ MDA-MB-231 cells was plated in each well of a 6-well plate, a day prior to plasmid transfection. The following day, the cells were transfected with 2 μg of pCMV6-AC-YBX1-GFP overexpression plasmid which encodes the ORF of *YBX1* gene (Origene, Rockville, MD, USA) or the pCMV6-AC-GFP empty vector control (Origene) using 6 μl of TurboFectin 8.0 (Origene) per well. The transfected cells were then incubated for 72 h for protein isolation. Experiments were performed in triplicate wells for each plasmid.

### Gaussia luciferase reporter assay

Gaussia luciferase reporter constructs containing the promoter region of *CORO1C* gene and a negative promoter region (both from Genecopoeia, Rockville, MD, USA) were used. 2.5 × 10^5^ MDA-MB-231 cells were seeded per well in a 6-well plate and co-transfected with 20 nM of *siNeg* or *siYB-1* with 1 μg of *CORO1C* promoter reporter construct, using Lipofectamine 2000 (Invitrogen). Also, 1 μg of negative promoter construct was used in place of the *CORO1C* promoter reporter construct and co-transfected with 20 nM of *siYB-1*. The co-transfections were done in triplicates. Media was changed 24 h post transfection. Subsequently, the cell culture supernatant was collected from each well 48 h post transfection and the Gaussia luciferase (GLuc) and secreted alkaline phosphatase (SEAP) activities were assessed using the Secrete-Pair Dual Luminescence Assay Kit (Genecopoeia), in triplicates with 1500 ms integration time. Normalised data (relative luminescence unit, RLU) was calculated as the ratio of GLuc/SEAP activities from the triplicate readings. The average RLU was calculated from an average of the normalised data for the triplicate transfected wells.

In addition, MDA-MB-231 cells were co-transfected with 1 μg of *CORO1C* promoter reporter construct and 1 μg of YB-1 ORF construct (pCMV6-AC-YBX1-GFP) or empty vector construct (pCMV6-AC-GFP) in a 6-well plate using TurboFectin 8.0 (Origene), in accordance with the manufacturer’s protocol. 1 μg of negative promoter construct was also used in place of *CORO1C* promoter reporter construct and co-transfected with 1 μg of YB-1 ORF plasmid. After which, the same procedure as mentioned above was repeated.

### Co-transfection of siRNA and plasmid DNA

6 × 10^4^ MDA-MB-231 cells were seeded in each well of a 24-well plate. The following day, cells were transfected with 200 ng of pCMV6-AC-GFP empty vector control (Origene) or *CORO1C* ORF construct (pCMV6-AC-CORO1C-GFP) (Origene) with 1μl of Lipofectamine 3000 and 0.8μl of P3000 (Invitrogen), according to the manufacturer’s protocol and incubated overnight. Subsequently, the cells were transfected with *siNeg* or *siYB-1* siRNA to a final concentration of 20 nM per well using DharmaFECT (GE Dharmacon) as described above. The cells were incubated for an additional of 48 h before seeding for migration or invasion assay.

### Statistical analyses

The GraphPad Prism 5.0 was used for analyses. Data was analyzed with two-sided unpaired Student’s t-test for samples with two groups and One-way ANOVA for samples with more than two groups. All values are represented as mean ± SEM. The results were considered statistically significant when *p* is <0.05.

## Results

### Downregulation of YB-1 protein decreased cell migration and invasion

**Fig. 1:**
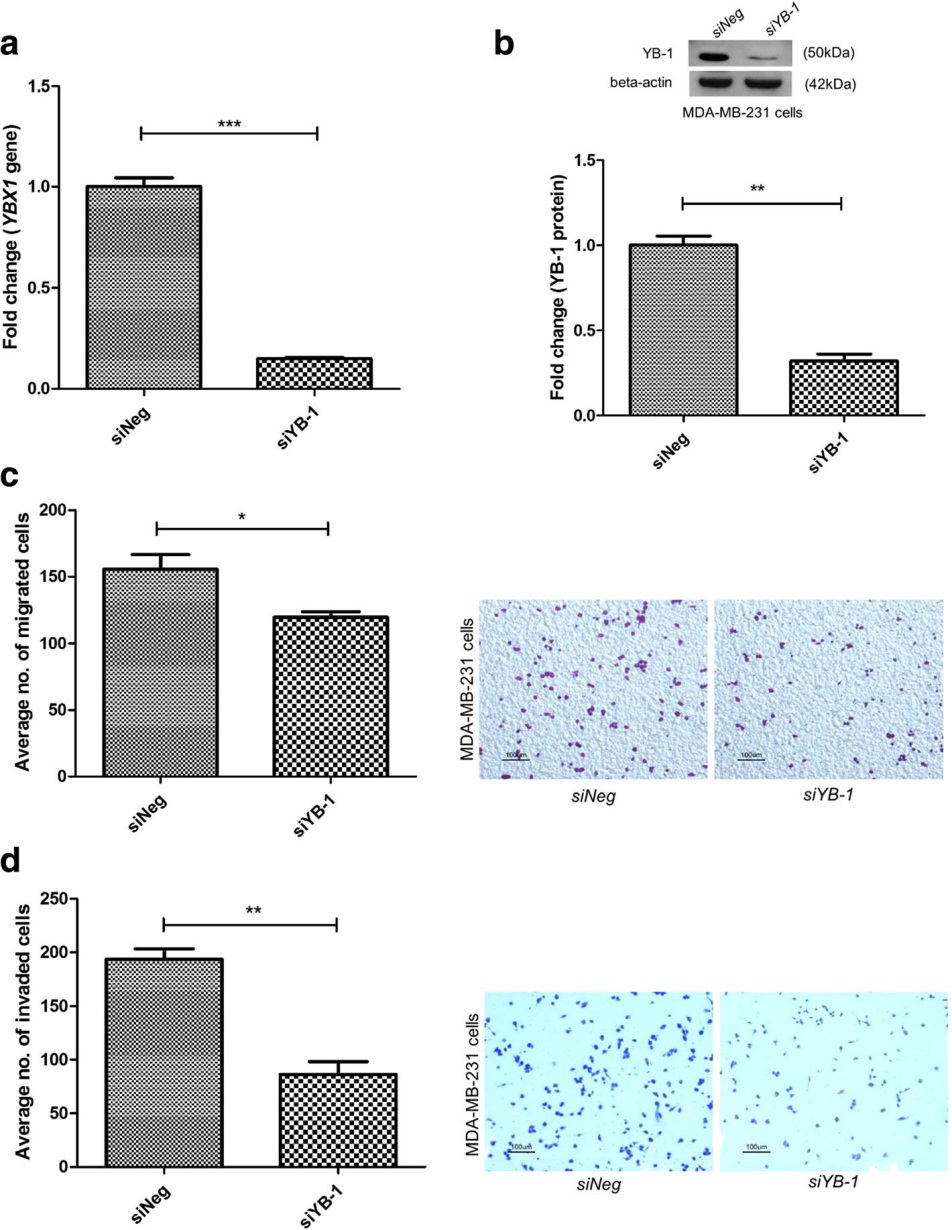
*YBX1* silencing in MDA-MB-231 cells reduced cell migration and cell invasion. **a** *YBX1* gene expression in YB-1 silenced MDA-MB-231 cells. **b** YB-1 protein expression in YB-1 silenced MDA-MB-231 cells and a representative image of the Western blot. **c**-**d** MDA-MB-231 cells were transfected with siRNA and alterations in cell migration and invasion were determined using transwell inserts. Error bar = SEM, *p < 0.05, **p < 0.01, ***p < 0.001 indicates statistically significant difference. Representative fields of the migration and invasion assay at 10X magnification are shown (Scale bar =100 μm)

To evaluate YB-1 function in breast cancer metastasis, silencing of the *YBX1* gene by siRNA was carried out in MDA-MB-231 breast cancer cells. The silencing effciency of the *YBX1* gene was approximately 85.1% in the *siYB-1* cells (Fig. 1a), while at the protein level, YB-1 protein was reduced by approximately 68% (Fig. 1b). A significant decrease in cell migration (approximately 21.2%) (Fig. 1c) and cell invasion (55.5%) (Fig. 1d) were observed in the *YBX1* silenced MDA-MB-231 cells when compared to control (*siNeg*) cells.

**Fig. 2:**
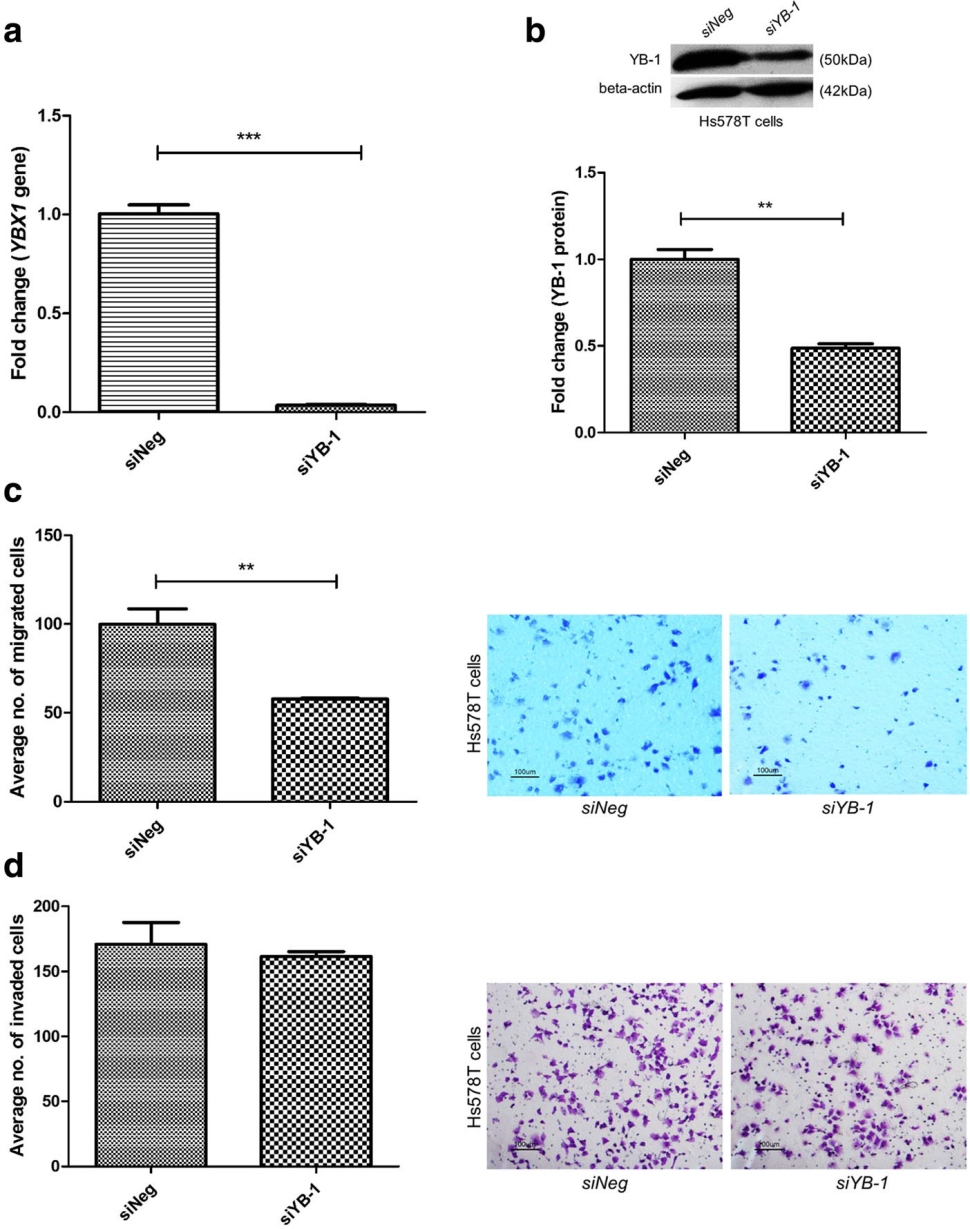
*YBX1* silencing in Hs578T cells inhibited cell migration but not cell invasion. **a**-**b** Knockdown of *YBX1* in Hs578T breast cancer cells decreased gene level and protein expression of YB-1. **c**-**d** *YBX1* silenced cells showed a a concomitant reduction in cell migration and a downward trend for cell invasion. Error bar = SEM, ** *p* < 0.01, ***p < 0.001. Representative fields of the migration and invasion assay at 10X magnification are shown as well (Scale bar =100 μm)

The findings were also validated in invasive Hs578T breast cancer cells. The YB-1 transcript and protein level were successfully down-regulated following transfection with siRNA (Fig. 2a and b). There was a concomitant and significant decline in cell migration (42.3%) (Fig. 2c), but not cell invasion (Fig. 2d) when compared to *siNeg* cells, although a downward trend was observed for cell invasion upon *YBX1* silencing.

### Global gene expression profiling identified *CORO1C* as a potential downstream target of YB-1

**Fig. 3:**
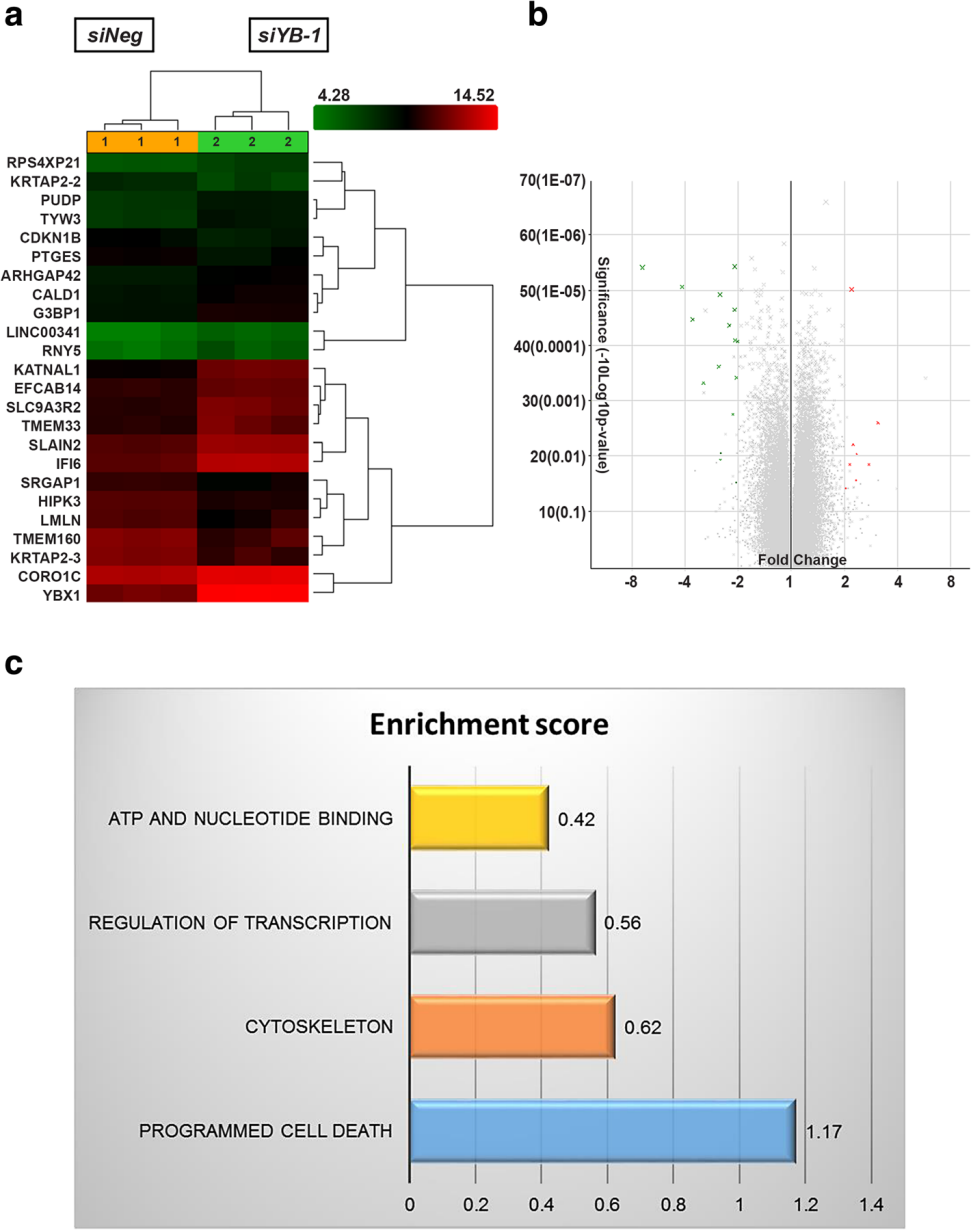
Global gene expression profiling in YB-1 silenced MDA-MB-231 cells. Gene expression profiling was carried out using Affymetrix GeneChip® Human Transcriptome 2.0 Array. **a** Hierarchical clustering and **b** Volcano plot of the differentially expressed genes using a cut-off value of *p* < 0.05 and fold change <−2 and >2. **c** Enrichment score of functional annotation clustering using DAVID analysis

To identify potential targets of the YB-1 protein, and to unravel the molecular mechanism underlying the observed phenomena upon silencing of the *YBX1* gene, global gene expression profiling using the Affymetrix GeneChip® Human Transcriptome 2.0 Array was carried out in the *YBX1* silenced MDA-MB-231 cells. Using a cut off value of <−2 and >2 and *p* value of <0.05, a total of 8 coding genes were found to be up-regulated and 12 coding genes (excluding *YBX1*) were down-regulated in the *siYB-1* MDA-MB-231 cells when compared to *siNeg* cells (Fig. 3a and b), which included both the annotated non-coding and coding genes. Table 1 shows the list of annotated coding genes that were differentially expressed*.* Additional file 2: Table S2 shows three non-coding genes that were differentially expressed.

**Table 1:** List of differentially expressed annotated coding genes

Down-regulated genes			Up-regulated genes		
Gene symbol	Fold change (*siYB-1 vs. siNeg*)	*p* value	Gene Symbol	Fold change (*siYB-1 vs. siNeg*)	*p* value
*YBX1*	-6.99	0.000004	*PTGES*	2.04	0.038408
*KATNAL1*	-4.16	0.000009	*CDKN1B*	2.15	0.014217
*IFI6*	-3.63	0.000034	*HIPK3*	2.2	0.00001
*SLC9A3R2*	-3.15	0.000483	*SRGAP1*	2.24	0.006231
*TYW3*	-2.58	0.000243	*LMLN*	2.33	0.027208
*SLAIN2*	-2.54	0.000012	*KRTAP2–2*	2.34	0.009131
*TMEM33*	-2.52	0.008867	*TMEM160*	2.75	0.014184
*PUDP*	-2.25	0.000043	*KRTAP2–3*	3.1	0.00254
*CORO1C*	-2.1	0.000023			
*G3BP1*	-2.09	0.000004			
*EFCAB14*	-2.09	0.000081			
*CALD1*	-2.05	0.000385			
*ARHGAP42*	-2.02	0.000085			

To validate the accuracy of the microarray data, expression of the highest differentially expressed genes was quantitated by qPCR. All the genes analyzed showed a consistent pattern with the microarray results (Additional file 3: Figure S1a), suggesting that the microarray data was highly reliable and reproducible. DAVID analysis of the differentially regulated genes identified these genes as highly enriched for cytoskeleton (Fig. 3c), suggesting that YB-1 protein could participate in the regulation of cytoskeletal structures, leading to changes in cell migration or invasion, which corroborates with the results obtained.

**Fig. 4:**
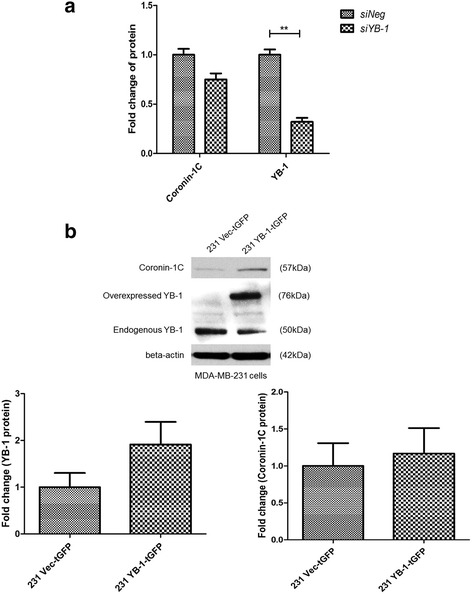
Coronin-1C protein expression is dependent on YB-1 protein expression. **a** Coronin-1C protein expression was decreased when YB-1 was knocked down in MDA-MB-231 cells. **b** Coronin-1C protein expression was increased when YB-1 protein was overexpressed in MDA-MB-231 cells. Error bar = SEM, **p < 0.01, indicates statistically significant difference. Representative samples of Western blots are shown as well

**Fig. 5:**
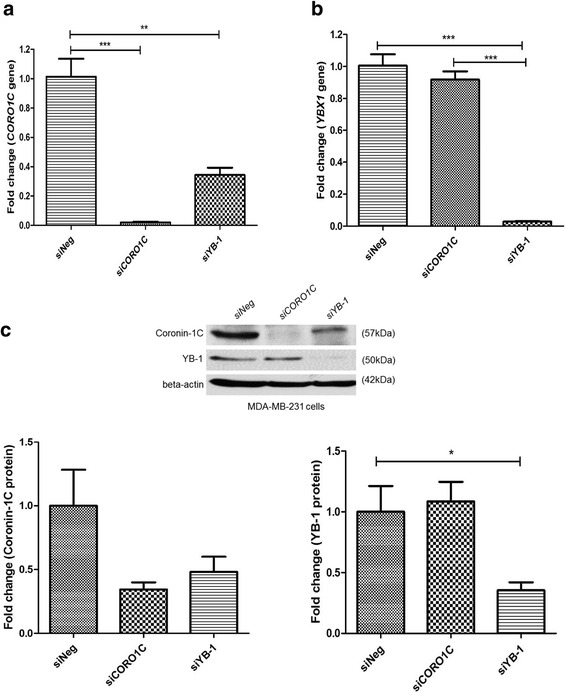
Coronin-1C is a downstream target of YB-1. **a** Gene expression of *CORO1C* upon silencing of *YBX1* or *CORO1C*. **b** Gene expression of *YBX1* upon silencing of *YBX1* or *CORO1C*. **c** Representative images of Western blot showing the expression of coronin-1C and YB-1 proteins. There is a decrease in *CORO1C* gene or protein expression upon silencing of *CORO1C* or *YBX1* when compared to *siNeg* cells in MDA-MB-231 cell line. In contrast, *YBX1* gene and protein expression were decreased in *siYB-1* MDA-MB-231 cells only, indicating that coronin-1C is a likely downstream target of YB-1. Error bar = SEM, *p < 0.05, **p < 0.01, ***p < 0.001, indicates statistically significant difference

Subsequently, the protein expression of some differentially expressed genes, which have been shown to be involved in cancers and may play a role in migration and invasion in breast cancer, was screened by Western blot in *YBX1* silenced MDA-MB-231 cells (Additional file 3: Figure S1b), and coronin-1C was then selected for further investigation. The protein expression of coronin-1C was found to be decreased when YB-1 was silenced (Fig. 4a and Additional file 3: Figure S1b). Conversely, YB-1 overexpression in the MDA-MB-231 cells induced a modest increase in coronin-1C expression (Fig. 4b), suggesting that coronin-1C is likely to be a downstream target of YB-1 protein. While down-regulation of YB-1 alone reduced expression of the *CORO1C* gene and protein concomitantly (Fig. 5a and c), knockdown of *CORO1C* alone did not alter the gene and protein expression of YB-1 (Fig. 5b and c), further validating that coronin-1C is a downstream target of YB-1.

**Fig. 6:**
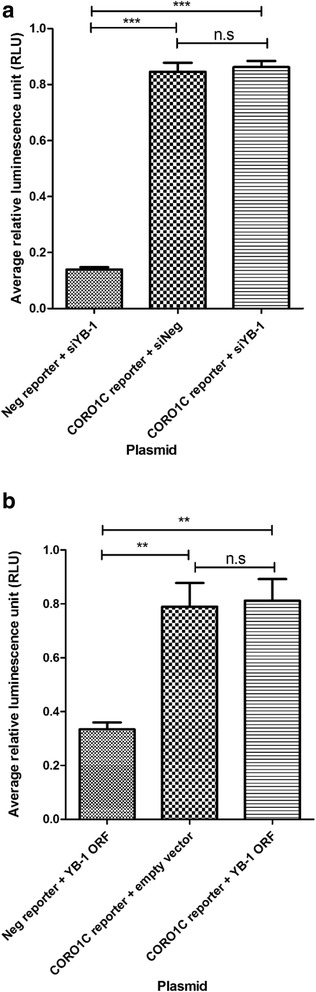
*CORO1C* is an indirect target of YB-1. **a** Gaussia luciferase reporter assay revealed no significant difference in the average relative luminescence signal (RLU) between cells co-transfected with *CORO1C* promoter construct and *siNeg* or *siYB-1* cells*.* **b** Gaussia luciferase reporter assay showed no significant difference in average RLU between the cells co-transfected with *CORO1C* promoter construct and YB-1 ORF plasmid or empty vector backbone. These results indicate that YB-1 does not bind directly to the *CORO1C* promoter. Error bar = SEM, **p < 0.01, ***p < 0.001, indicates statistically significant difference

To determine whether YB-1 directly regulates *CORO1C* at the transcriptional level, the Gaussia luciferase reporter assay was performed. However, there was no reduction in luciferase signal after YB-1 silencing (Fig. 6a). The RLU signal was found to be significantly increased upon co-transfection of the *CORO1C* promoter reporter construct with both the YB-1 ORF plasmid or empty vector backbone in contrast to co-transfection of the negative promoter construct with the YB-1 ORF plasmid in MDA-MB-231 cells (Fig. 6b). Hence, the results suggest that YB-1 does not regulate transcription of *CORO1C* in a direct manner.

**Fig. 7:**
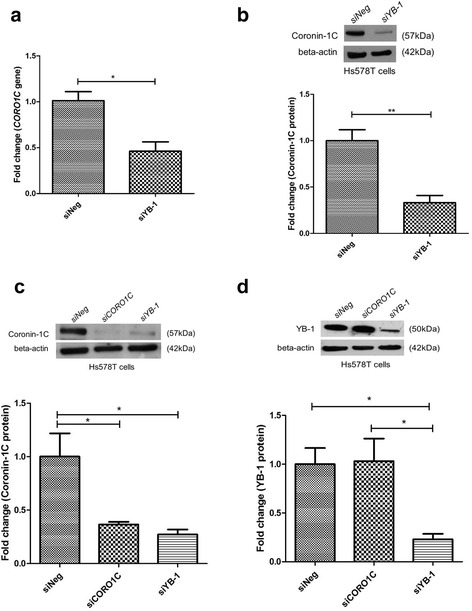
YB-1 silencing decreased coronin-1C expression in the highly invasive Hs578T breast cancer cell line. **a**-**b** *CORO1C* gene level and protein expression of coronin-1C decreased when *YBX1* was knocked down in Hs578T cells. **c**-**d** Protein expression of coronin-1C and YB-1 in either *CORO1C* or *YB-1* silenced Hs578T cells. Error bar = SEM, *p < 0.05, **p < 0.01, indicates statistically significant difference

The same biological effect was also elicited in Hs578T cells, whereby silencing of the *YBX1* gene in Hs578T cells also resulted in a concomitant decrease in the gene (54.3%) and protein expression of coronin-1C (66.9%) (Fig. 7a–c) but knockdown of *CORO1C* alone did not alter the protein expression of YB-1 (Fig. 7d).

### siRNA- mediated silencing of *CORO1C* inhibits cell migration and invasion

**Fig. 8:**
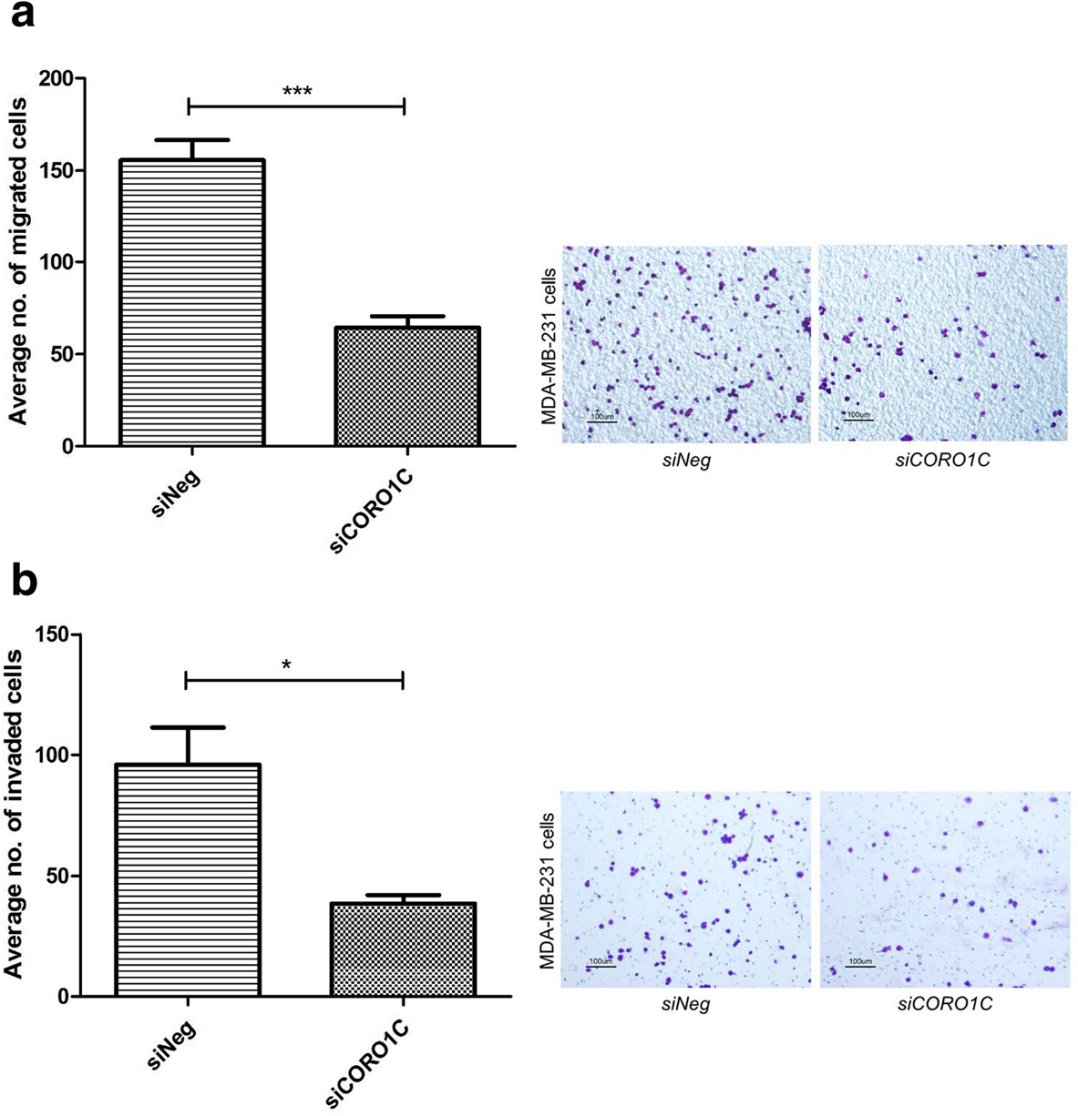
*CORO1C* silencing in MDA-MB-231 cells reduced cell migration and invasion. **a**-**b** Cell migration and invasion were significantly inhibited in the *siCORO1C*-treated MDA-MB-231 cells. Error bar = SEM, *p < 0.05, ***p < 0.001, indicates statistically significant difference. Representative fields of the migration and invasion assay at 10X magnification are shown as well (Scale bar =100 μm)

**Fig. 9:**
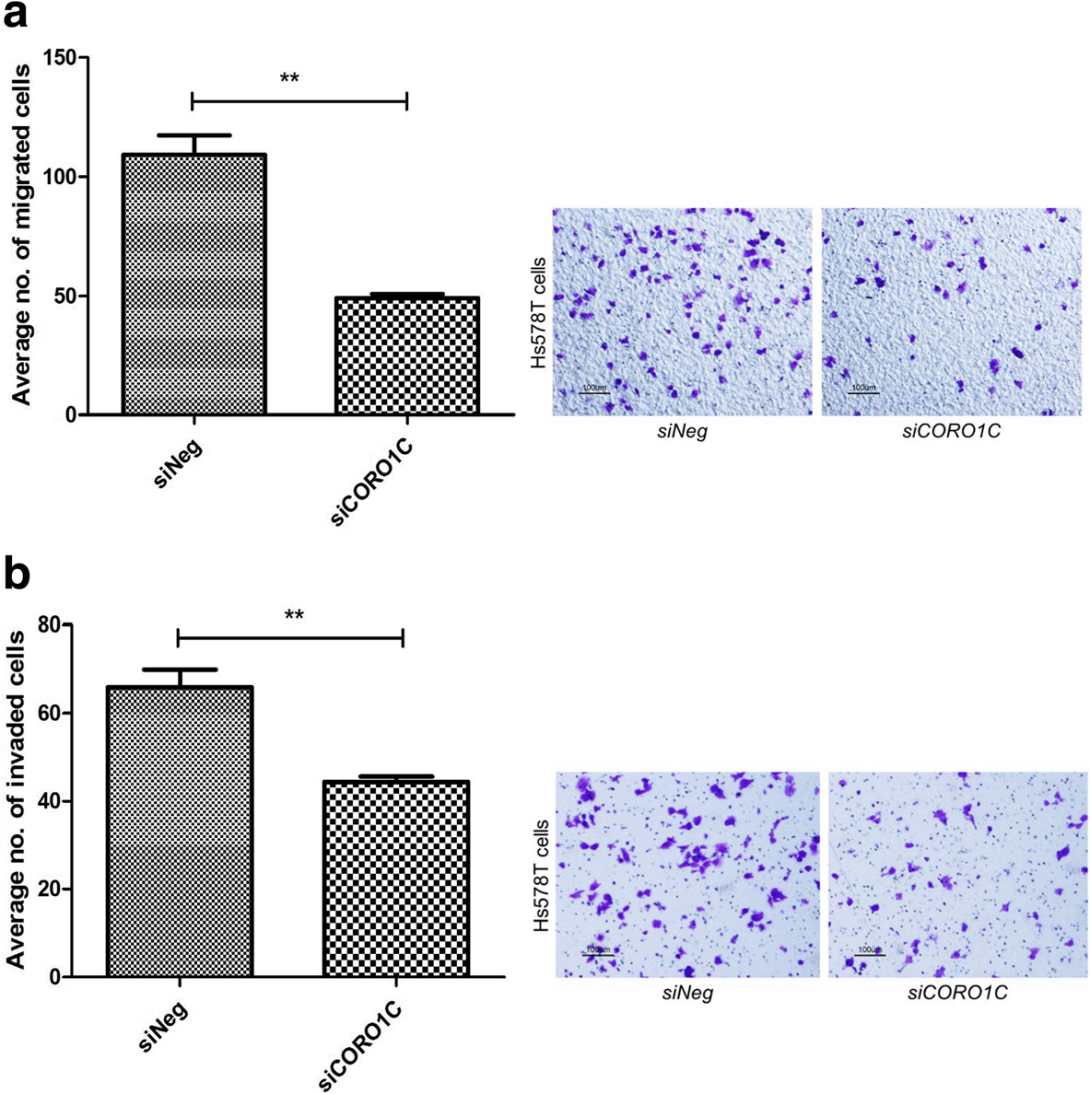
*CORO1C* silencing in Hs578T cells inhibited cell migration and invasion. **a**-**b** Cell migration and invasion were significantly reduced in si*CORO1C*-treated Hs578T cells. Error bar = SEM, **p < 0.01. Representative fields of the migration and invasion assay at 10X magnification are shown as well (Scale bar =100 μm)

Silencing of *CORO1C* induced a significant decrease in cell migration (52.6%) (Fig. 8a) and cell invasion (63.9%) (Fig. 8b) in MDA-MB-231 breast cancer cells. Similar findings were also observed in *siCORO1C*-treated Hs578T cells (Fig. 9).

### siRNA-mediated YB-1- silencing in *CORO1C* overexpressing cells decreased cell migration and invasion

**Fig. 10:**
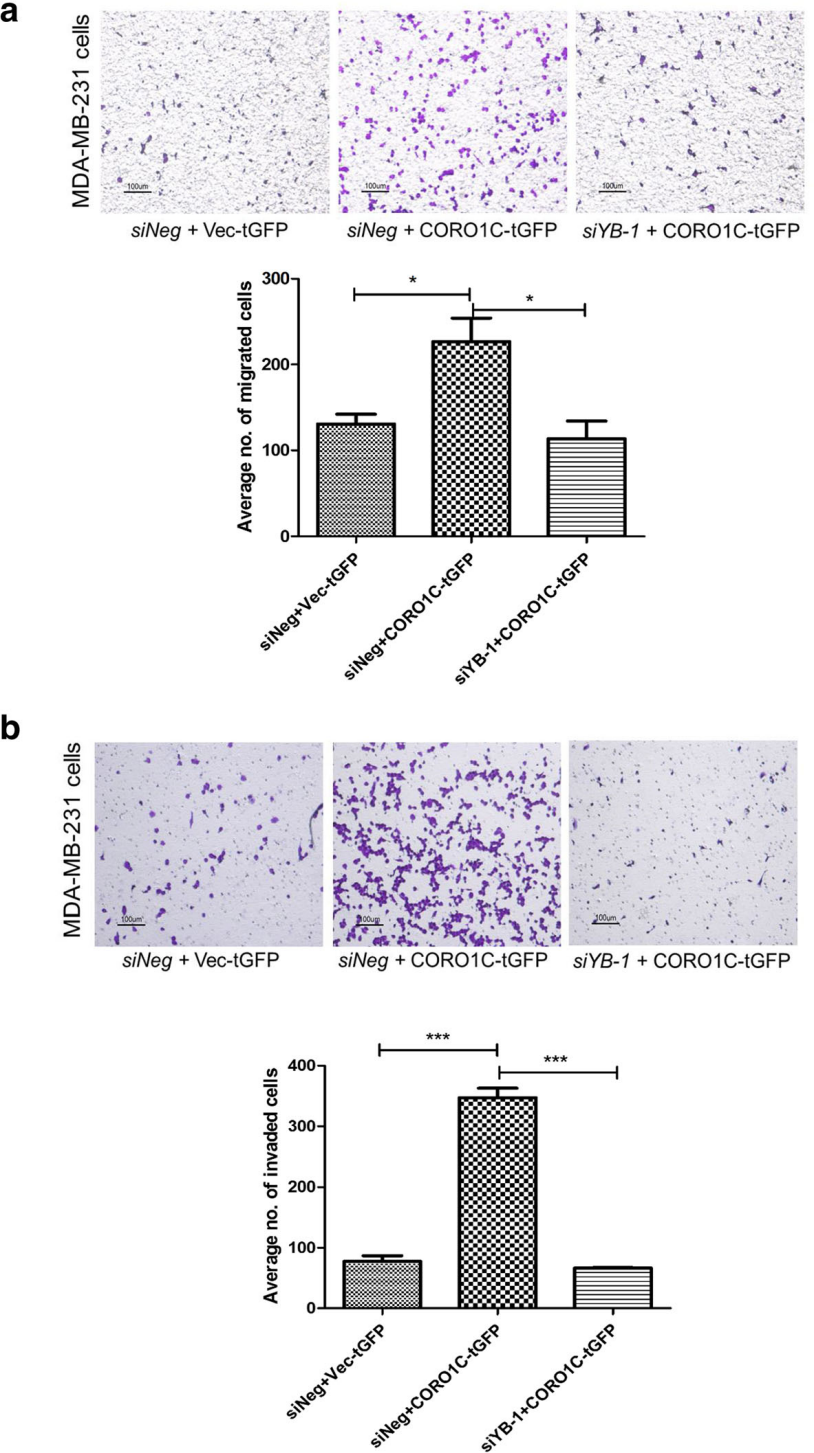
**a**-**b** Silencing of *YBX1* gene in coronin-1C overexpressing MDA-MB-231 cells decreased cell migration and invasion. Error bar = SEM, *p < 0.05*,* ***p < 0.001. Representative fields of the migration and invasion assay at 10X magnification are shown (Scale bar =100 μm)

To further substantiate the notion that YB-1 could regulate cell migration and invasion through coronin-1C, a rescue experiment was carried out by silencing *YBX1* in *CORO1C* overexpressing MDA-MB-231 cells. Coronin-1C overexpression induced a marked increase in cell migration and invasion (Fig. 10), which were attenuated by the knockdown of YB-1 in the *CORO1C* overexpressing cells.

## Discussion

Metastasis, the leading cause for cancer related deaths, is regulated by diverse molecular pathways in cancer cells and involves a complex multi-step process collectively termed as the ‘invasion-metastasis cascade’ [2]. YB-1 has been found to promote a metastatic phenotype in breast cancer [17], gastric cancer [7, 18] and prostate cancer [19]. Moreover, YB-1 has been suggested to be an independent predictor of liver metastasis and relapse in patients with advanced gastric cancer [18]. In addition, YB-1 is overexpressed in the majority of triple-negative breast cancers (TNBC), which belongs to a highly aggressive subtype of breast cancer [20]. Hence, to obtain a better understanding of the functional role(s) of YB-1 in breast cancer metastasis, the *YBX1* gene was knocked down in two highly aggressive TNBC cell lines, namely MDA-MB-231 and Hs578T cells. Knockdown of YB-1 in MDA-MB-231 cells led to a decrease in both the migration and invasive potential of the breast cancer cells. Our results are in line with a previous study where YB-1 was observed to mediate migratory and invasive properties of MDA-MB-231 cells [17].

Gene expression profiling in *YBX1* silenced MDA-MB-231 cells was performed to identify potential targets of YB-1 that are important in metastasis. Interestingly, from the functional annotation clustering of the differentially expressed genes, cytoskeleton proteins were found to be highly enriched. One of the fundamental steps for metastasis is the ability of cancer cells to migrate or invade, which is driven by actin re-modelling, formation of protrusions and cell adhesion that are mediated by several proteins [21]. Actin re-modelling is mediated by several proteins such as N-WSAP, Arp2/3 complex, cofilin and coronins [22, 23]. The role of actin re-modelling and its regulators in cancer metastasis has been widely studied [24, 25]. Nevertheless, much remains to be elucidated with regard to the exact mechanisms or genes that regulate actin re-modelling, resulting in acquisition of migratory or invasive abilities by cancer cells, a key step in cancer metastasis. Furthermore, the YB-1 protein has been observed to bind to actin and tubulin, and promote microtubule assembly [26]. YB-1 may therefore regulate different structural components of the cytoskeleton.

Our results showed that the expression of coronin-1C was dependent on the expression of YB-1 but not vice-versa (in both MDA-MB-231 cells and Hs58T cells), suggesting that coronin-1C is a likely downstream target of YB-1. Coronin-1C (also known as Coronin 3) is a member of the coronin family of actin-binding proteins that regulate re-modelling of actin filaments and thus control cell migration which relies on actin dynamics [27, 28]. Coronins are known to co-localise with F-actin [29]. They recruit Arp2/3 complex to ATP-F-actin, and inhibit cofilin, thus preventing disassembly at the fast growing ends (plus end) of cells, and facilitating assembly of actin filaments. However, coronins also recruit cofilin to the slow growing ends (minus end), with the opposite effect of favoring disassembly of actin filaments [30]. These mechanisms result in elongation of the cell membrane at the leading edge, and retraction of the cells at the rear-end, thereby facilitating cell migration and even invasion.

As we were interested in metastasis-related proteins, we focused on proteins highly enriched for cytoskeleton-related function as shown by the DAVID functional annotation analysis. Coronin-1C was selected for further investigation as this protein has been associated with metastasis in a variety of cancers, including breast cancer [31] and other malignancies such as lung [32], diffuse glioma [29], hepatocellular carcinoma [33] and gastric cancer [25]. Furthermore, expression of coronin-1C which is known to be highest in the TNBC subtype, has been reported to correlate with lower survival [31]. Recently, microRNA-206 (miR-206) was found to reduce cell migration in MDA-MB-231 and SUM159 TNBC cell lines, by regulating the expression of its target gene, coronin-1C [31]. Moreover, overexpression of coronin-1C was found to correlate with lymph node spread and increased clinical stage in gastric carcinoma, possibly *via* the regulation of MMP-9 and cathepsin K [25]. In diffuse glioma, silencing of coronin-1C led to reduced invadopodia formation, migration and invasion [24]. Taken together, these findings suggest the importance of elevated coronin-1C in tumour progression and metastasis. The other interesting differentially expressed genes that were found to be enriched for cytoskeleton-related function as analysed by DAVID in this present study, were *CALDI, KRTAP2–2* and *KRTAP2–3. CALDI*, which encodes for caldesmon, a versatile protein that binds to actin, myosin, calmodulin and tropomyosin, is involved in cell motility [34] and regulates metastasis of gastric cancer [35]. *KRTAP2–2* and *KRTAP2–3* belongs to the hair keratin–associated proteins (KRTAPs) family which form major structural components of the hair shaft with no known function in cancer cells [36]. Another differentially expressed gene identified, encodes the Rho GTPase activating protein, which is also known to be involved in the regulation of cell motility and cytoskeletal dynamics [37]. It may therefore be compelling to determine the association of YB-1 with these genes and their roles in breast cancer metastasis.

To the best of our knowledge, this is the first time that YB-1 has been demonstrated to drive breast cancer metastasis (cell migration and/or invasion) *via* coronin-1C. Silencing of YB-1 or coronin-1C in MDA-MB-231 cells resulted in decreased cell migration and cell invasion, suggesting that YB-1 regulates these cellular processes *via* coronin-1C. Moreover, we observed a similar phenotype upon *YBX1* silencing in the invasive Hs578T cells, including significant reduction in cell migration and a downward trend for cell invasion (although not significant). The latter observation could be due to the different gene expression profiles of the two cell lines as MDA-MB-231 cells are derived from an adenocarcinoma while Hs578T cells are derived from a carcinosarcoma [38]. In addition, the decrease in cell migration and invasion observed upon YB-1 silencing in coronin-1C overexpressed cells, further confirmed our hypothesis that YB-1 plays an important role in regulating cell migration and invasion through regulation of coronin-1C expression. The luciferase reporter assay revealed that coronin-1C is an indirect target of YB-1, suggesting that other intermediary proteins may also be involved. It is worth noting that some of the differentially expressed genes from the microarray analysis included non-coding genes such as long non-coding RNA. A previous report has found that non-coding RNAs such as microRNAs are directly involved in the transcriptional regulation of coronin-1C in breast cancer [31]. As non-coding genes were not part of the focus in this study, we did not further evaluate in-depth the roles of these genes. Several studies have also shown that silencing *CORO1C* decreases both cell invasion and migration [24, 25, 29, 32], which is consistent with our findings, thus suggesting its therapeutic and prognostic potential.

## Conclusion

In conclusion, the association between YB-1 and coronin-1C provides a novel pathway which could be a potential therapeutic target in breast cancer metastasis. Overexpression of YB-1 and coronin-1C observed in TNBC suggests the need for more comprehensive investigation of this association. Future identification of intermediate proteins that are involved in YB-1 regulated coronin-1C could provide further biological insights regarding the metastatic cascade, with the possibility of identifying reliable biomarkers of early metastasis.

## Additional files

Additional file 1: Table S1. Sequences of primers used for qPCR. (XLSX 12 kb)
Additional file 2: Table S2. Non-coding genes that were differentially expressed following YB-1 silencing in MDA-MB-231 cells. (XLSX 10 kb)
Additional file 3: Figure S1. a Selected genes were used to validate the microarray data analysis using qPCR and the expression of all of the genes showed consistent patterns when compared to the microarray data. b Protein expression of some differentially expressed genes were screened by Western blotting in *YBX1* silenced MDA-MB-231 cells and the representative blot is shown. (TIFF 403 kb)

## Abbreviations

siRNA: small interfering RNA
TNBC: triple-negative breast cancer
WHO: World Health Organisation
YB-1: Y-box binding protein-1
