# Supplementary figures and images for: YBX1 gene silencing inhibits migratory and invasive potential via CORO1C in breast cancer in vitro
Source: BMC Cancer. 2017 Mar 16;17:201. doi: 10.1186/s12885-017-3187-7 (PMC5356414; doi:10.1186/s12885-017-3187-7)

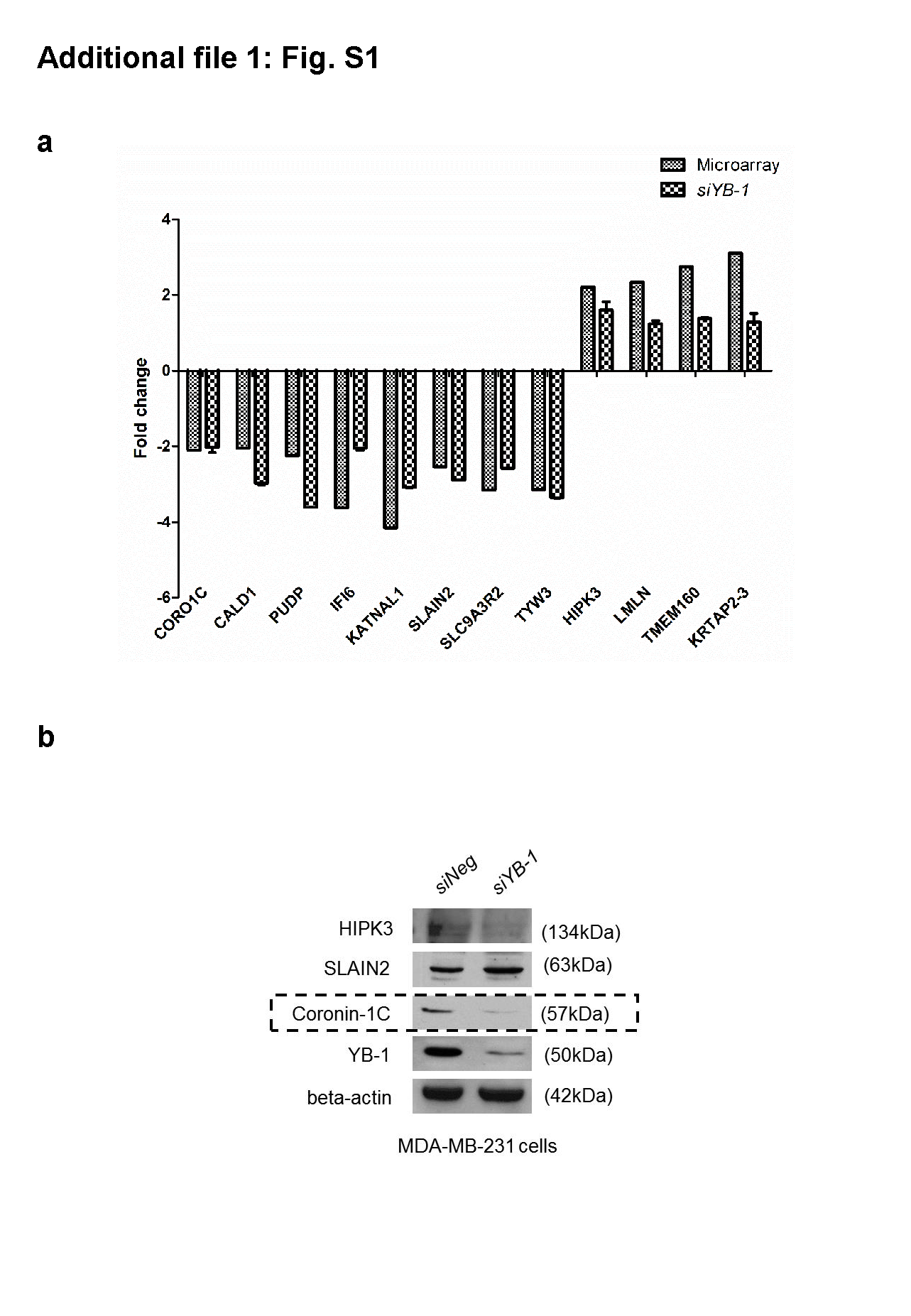

Supplement: Additional file 3: Figure S1. — a Selected genes were used to validate the microarray data analysis using qPCR and the expression of all of the genes showed consistent patterns when compared to the microarray data. b Protein expression of some differentially expressed genes were screened by Western blotting in YBX1 silenced MDA-MB-231 cells and the representative blot is shown. (TIFF 403 kb) [file 12885_2017_3187_MOESM3_ESM.tif]
